# Genomics of *Acinetobacter baumannii* iron uptake

**DOI:** 10.1099/mgen.0.001080

**Published:** 2023-08-07

**Authors:** Irene Artuso, Harsh Poddar, Benjamin A. Evans, Paolo Visca

**Affiliations:** ^1^​ Department of Science, Roma Tre University, Viale G. Marconi 446, 00146 Rome, Italy; ^2^​ Norwich Medical School, University of East Anglia, Rosalind Franklin Road, Norwich Research Park, Norwich, NR4 7UQ, UK; ^3^​ Fondazione Santa Lucia IRCCS, Via Ardeatina, 306/354, 00179 Rome, Italy; ^4^​ National Biodiversity Future Centre, Palermo 90133, Italy

**Keywords:** *Acinetobacter baumannii*, haem, iron uptake, phylogeny, sequence type, siderophore

## Abstract

Iron is essential for growth in most bacteria due to its redox activity and its role in essential metabolic reactions; it is a cofactor for many bacterial enzymes. The bacterium *Acinetobacter baumannii* is a multidrug-resistant nosocomial pathogen. *A. baumannii* responds to low iron availability imposed by the host through the exploitation of multiple iron-acquisition strategies, which are likely to deliver iron to the cell under a variety of environmental conditions, including human and animal infection. To date, six different gene clusters for active iron uptake have been described in *A. baumannii *, encoding protein systems involved in (i) ferrous iron uptake (*feo*); (ii) haem uptake (*hemT* and *hemO*); and (iii) synthesis and transport of the baumannoferrin(s) (*bfn*), acinetobactin (*bas*/*bau*) and fimsbactin(s) (*fbs*) siderophores. Here we describe the structure, distribution and phylogeny of iron-uptake gene clusters among >1000 genotypically diverse *A. baumannii* isolates, showing that *feo*, *hemT*, *bfn* and *bas*/*bau* clusters are very prevalent across the dataset, whereas the additional haem-uptake system *hemO* is only present in a portion of the dataset and the *fbs* gene cluster is very rare. Since the expression of multiple iron-uptake clusters can be linked to virulence, the presence of the additional haem-uptake system *hemO* may have contributed to the success of some *A. baumannii* clones.

## Data Summary

All genome sequences used in this study were downloaded from the National Center for Biotechnology Information (NCBI) database or from the PubMLST database and are listed in Dataset S1. The Supplementary Material has been uploaded to Microbiology Society figshare: https://doi.org/10.6084/m9.figshare.23629152.v1 [1].

**Impact Statement**

Iron plays a crucial role in bacterial physiology and pathogenicity, and iron acquisition systems have become attractive targets for the development of new antibacterial agents against multidrug-resistant bacteria, including *Acinetobacter baumannii *. This bacterium exploits multiple systems for the acquisition of different iron forms, which are variably combined in the global *A. baumannii* population. In this work, we illustrate the distribution of iron-uptake gene clusters across a large dataset of *A. baumannii* genomes, representative of the main lineages circulating worldwide. Our findings provide valuable insights into the prevalence, diversity and evolution of iron-uptake systems in *A. baumannii *, which may help explain its success as a pathogen. The results of this study may also aid in the development of novel therapies against *A. baumannii* infections.

## Introduction

*Acinetobacter baumannii* has emerged as an important opportunistic pathogen worldwide [2]. Antibiotic resistance is widespread in *A. baumannii *, leading the World Health Organization (WHO), the US Food and Drug Administration (FDA) and the US Centers for Disease Control (CDC) to classify this bacterium as one of the most critical pathogens for which new antimicrobials should urgently be developed [3–5]. *A. baumannii* causes multidrug- and even pandrug-resistant nosocomial infections in critically ill patients, frequently manifesting as bloodstream infections and ventilator-associated pneumonia [6]. Since the 1980s, the vast majority of *A. baumannii* outbreaks worldwide have been caused by strains belonging to two major clonal lineages (or clones), known as global clones 1 and 2, although new lineages are now common in some parts of the world [7–11]. In fact, particular sequence types (STs) determined by the multilocus sequence typing (MLST) Pasteur scheme, namely ST2 (belonging to the global clone 2), ST1 (belonging to the global clone 1), ST79, and ST25, account for over 71 % of all genomes sequenced to date, with ST2 by far the most dominant type in most countries [10]. ST2 is characterized by a multidrug-resistant (MDR) phenotype that has expanded alarmingly over the years, with frequent reports of *A. baumannii* strains resistant to almost all clinically relevant antibiotics [9, 12, 13]. Resistance to an extensive range of antibiotics, including last-resort agents such as carbapenems and even colistin (a last-resort, lifesaving drug), is a hallmark of *A. baumannii* infection and poses a serious therapeutic challenge to clinicians [14, 15].

Iron is essential for the growth of nearly all bacteria due to its redox activity and its role in many vital metabolic reactions. To defend from invading pathogens, mammals and birds have evolved sophisticated mechanisms to sequester iron during bacterial infection, through a process called nutritional immunity [16]. One key to the success of *A. baumannii* as a nosocomial pathogen can be found in the multiplicity of iron-acquisition mechanisms for scavenging this scant but essential nutrient *in vivo* [17]. Broadly, bacteria respond to low iron availability through the exploitation of multiple iron-acquisition strategies, including (i) the uptake of Fe^2+^ eventually generated by the reduction of Fe^3+^, (ii) the uptake of haem from host haemoproteins and (iii)*,* the synthesis and the secretion of low-molecular-weight iron-chelating compounds, called siderophores [18, 19], and the uptake of exogenous iron complexes, e.g. those formed with heterologous siderophores (xenosiderophores).

In Gram-negative bacteria, Fe^2+^ is acquired by the Feo system, encoded by the *feoABC* operon [20]. Extracellular Fe^2+^ can diffuse into the periplasm (i.e*.,* the space between the outer and inner membranes of Proteobacteria) via the porins located in the outer membrane, including the iron-regulated porin OmpW [21, 22]. The inner membrane protein FeoB is the permease involved in the transport of Fe^2+^ across the inner membrane by a GTP-driven active transport mechanism. FeoB is a large protein with a cytosolic N-terminal G-protein domain and a C-terminal integral inner membrane domain; FeoA is probably required to enhance the GTPase activity of FeoB; and FeoC is a small cytosolic protein likely acting as a transcriptional repressor (Fig. S1, available in the online version of this article) [23, 24].

High-affinity iron-uptake systems are invariably characterized by the presence of specific receptors on the outer membrane that are activated by a tripartite protein complex (TonB, ExbB and ExbD) spanning from the cytoplasmic to the outer membrane, called the ‘TonB complex’. The TonB complex converts the transmembrane proton gradient into the energy required to internalize the iron carrier (siderophores, haemophores, etc.) into the periplasm by opening a gated channel in the outer membrane receptor. Once in the periplasm, transport of the iron carrier across the inner membrane is mediated by an ABC permease [25] (Fig. S2). *A. baumannii* carries three *tonB* orthologues, but only one of them (*tonB3*) is essential for *A. baumannii* growth in low-iron media and human serum and contributes to virulence in animal models of infection [26].

**Fig. 1.**
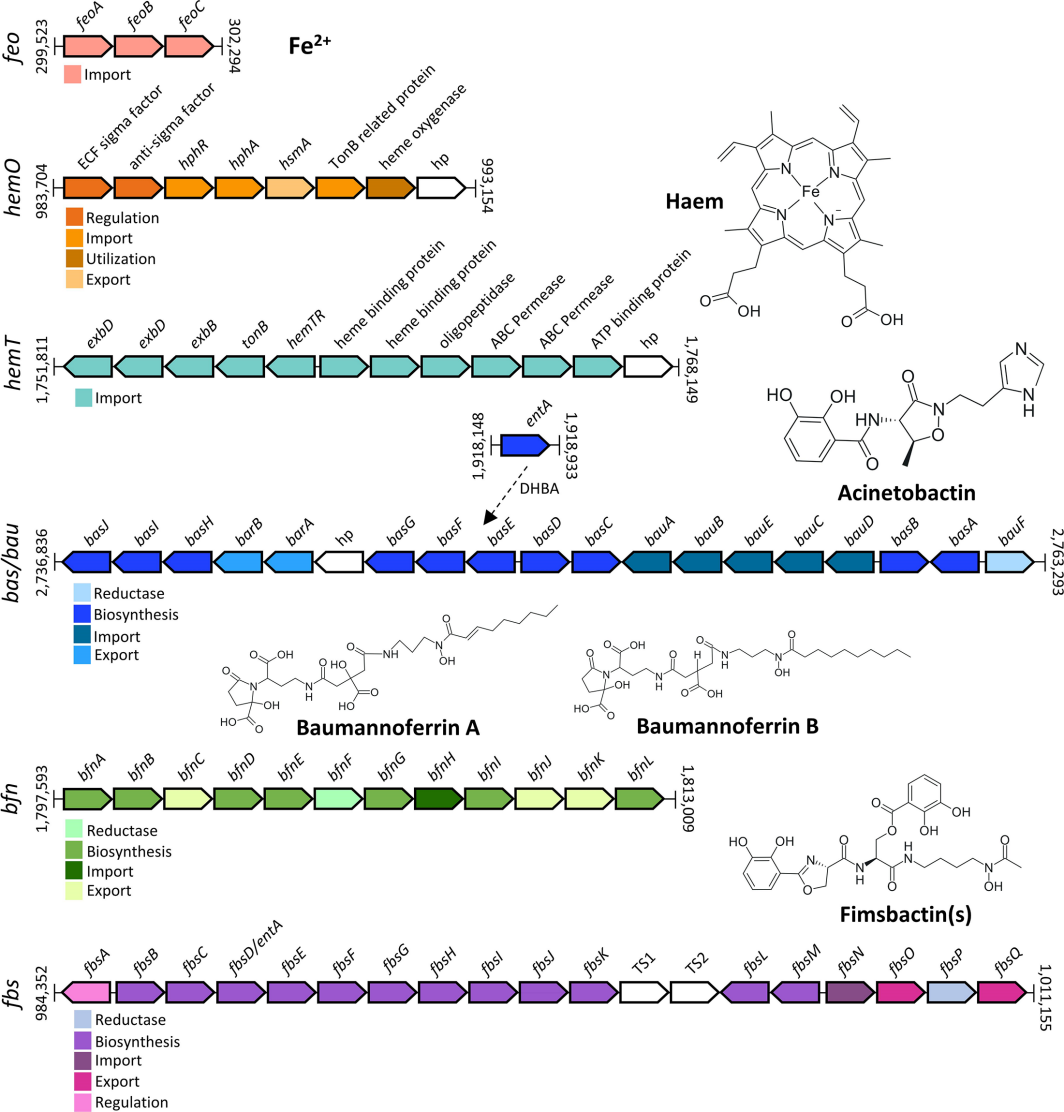
Genetic structure of iron-uptake gene clusters in *A. baumannii *. Gene annotations and positions refer to the genome of *A. baumannii* ACICU (NZ_CP031380.1), except for the fimsbactin(s) gene cluster, which refers to *A. baumannii* ATCC 17978 (NZ_CP012004.1). Different colours denote different gene functions. Genes with unknown function are shown in white. hp, hypothetical protein.

To date, six different gene clusters for active iron uptake have been described in *A. baumannii *, encoding protein systems involved in (i) the active transport of Fe^2+^ through the cytoplasmic membrane (*feo* gene cluster); (ii) haem uptake by producing specific transport systems for haem and haem-binding proteins (*hemT* and *hemO* gene clusters); and (iii) synthesis and transport of baumannoferrin(s) (*bfn* gene cluster), acinetobactin (*bas*/*bau* gene cluster) and fimsbactin(s) (*fbs* gene cluster) siderophores, which bind extracellular Fe^3+^ and actively transport metal into the cell (Fig. 1) [27]. The *feo* system is a universally conserved ferrous iron acquisition system in *A. baumannii* and represents the only proven system for direct Fe^2+^ uptake [26]. The most important iron-uptake molecule characterized in *A. baumannii* is the acinetobactin siderophore, which consists of equimolar quantities of 2,3-dhydrobenzoic acid, threonine and N-hydroxyhistamine and appears to be highly conserved among clinical isolates [28–30]. All the genes required for the biosynthesis (*basA-J*), efflux (*barAB*) and uptake (*bauA-E*) of acinetobactin are encoded from the same genomic locus, with the exception of an *entA* homologue, which is essential for the biosynthesis of the acinetobactin precursor 2,3-dihydroxybenzoic acid (DHBA), mapping to a separate location in the chromosome (Fig. 1) [31]. Acinetobactin is the only high-affinity iron-uptake system that is functional in *A. baumannii* ATCC19606^T^ [32], and it seems to play an essential role in *A. baumannii* pathogenicity [33].

Another important siderophore of *A. baumannii* is baumannoferrin, which was detected in several *A. baumannii* clinical isolates [27]. Structural analyses identified two isoforms, baumannoferrin A and baumannoferrin B, which only differ by a double bond and are composed of citrate, 1,3-diaminopropane, 2,4-diaminobutyrate, decenoic acid and α-ketoglutarate [34, 35]. Twelve genes are involved in the biosynthesis (*bfnA*, *B*, *D*, *E*, *G*, *I,* and *L*), transport (*bfnC*, *H*, *J*, and *K*) and utilization (*bfnF*) of baumannoferrin(s) (Fig. 1). Notably, the pathogenic *A. baumannii* strain AYE, which does not produce acinetobactin or other siderophores, proliferates robustly under iron limitation [34]. This suggests that the production of baumannoferrin(s) plays an important role in the fitness and pathogenicity of *A. baumannii *.

The third iron-chelating agent produced by *A. baumannii* is a mixed chatechol/hydroxamate siderophore called fimsbactin(s), which has so far been detected in a few strains, including *A. baumannii* strain ATCC 17978 originally isolated from fatal meningitis [27, 36]. The fimsbactin(s) locus (*fbsA-Q*) is flanked by transposases, suggesting that it may have been acquired by horizontal gene transfer [37]. Interestingly, the *fbsD* gene is an *entA* orthologue, and it can complement *entA* in *A. baumanni* strains lacking a functional *entA* copy (Fig. 1) [31].

*A. baumannii* was predicted to exploit two transport systems for the acquisition of haem from host haem reservoirs: the putative *hemT* system, which is present in almost all *A. baumannii* strains, and the *hemO* system, found in roughly two-thirds of clinical epidemic isolates [27]. Both haem-uptake clusters contain a unique TonB-dependent outer membrane receptor (HemTR and HphR). The *hemO* locus encodes the haem-degrading enzyme, haem oxygenase, and includes a gene coding for a haem scavenger (HphA), which is secreted by a surface lipoprotein assembly modulator (Slam) protein (HsmA) [38]. The *hemT* cluster encodes a periplasmic haem-binding protein and an inner membrane ATP-binding cassette (ABC) transporter (Fig. 1) [39]. However, the implication of the *hemT* cluster in haem uptake has not yet been experimentally confirmed.

The complexity that characterizes the *A. baumannii* iron-uptake mechanisms probably reflects the exceptional adaptability of this organism to face iron limitation in different environmental niches, including the human host. Multiple siderophores are thought to be important for avoiding siderophore piracy by other bacteria, facilitating iron acquisition under different physiological conditions and/or iron source composition, and possibly promoting the uptake of metals other than iron. It was demonstrated that all three siderophore biosynthetic pathways contribute to iron chelation *in vitro* and functional redundancy exists in their ability to facilitate iron mobilization from host sources. However, acinetobactin is essential for iron acquisition *in vivo*, whereas other siderophores, the baumannoferrin(s) and fimsbactin(s), appear to be dispensable [29]. Multiple haem-acquisition systems may also enable *A. baumannii* to use a broader range of host haem concentrations, and fine-tune gene expression. As such, *hemO* would enable *A. baumannii* to acquire haem when the pathogen first invades the bloodstream [38]. Given the crucial role of iron in bacterial physiology and pathogenicity, iron uptake has become an attractive target for the development of new antibacterial agents against MDR bacteria, including *A. baumannii* [40, 41].

Since previous *in silico* studies have shown that genotypically diverse *A. baumannii* isolates carry different iron-uptake systems [27, 39], the emergence of some epidemic lineages and strains over others could be the result of an improved ability to survive the extreme iron limitation (i.e. the so-called nutritional immunity) imposed by the host. While iron-uptake clusters have been identified and functionally characterized in a limited number of *A. baumannii* strains, no studies have so far been performed on a large-scale dataset. In recent years, the number of sequenced *A. baumannii* genomes, especially those of MDR clinical isolates, has increased significantly, so the present study aims to gain further insights into the distribution of *A. baumannii* iron-uptake gene clusters on a global scale using a large and representative dataset composed of the most prevalent lineages, which may help explain their emergence and success as human pathogens.

## Methods

### *A. baumannii* dataset selection

A complete list of *A. baumannii* genomes was retrieved from the MLST (Pasteur) database (updated 24 September 2021), from which only whole-genome sequences with a total length (Mbp) >=3.4 and a number of contigs <200, were selected (https://pubmlst.org/organisms/acinetobacter-baumannii). Redundant genomes were trimmed from the dataset, and only the last released genome for each strain was selected. Furthermore, all sequences that lacked ST assignment as well as isolates with a species assignment different from *A. baumannii* were removed. The resulting dataset includes 1071 whole-genome sequences (Dataset S1). A minimum spanning tree was generated from the MLST (Pasteur) allelic profiles of 1071 genome assemblies of *A. baumannii* using the plugin tool provided in the PubMLST database and visualized using GrapeTree [42].

### Iron-uptake gene sequences used in this study

The sequence of genes in the *feo, hemO, hemT*, *bas/bau* and *bfn* clusters and the *entA* gene of *A. baumannii* ACICU were used as references to investigate cluster and/or gene distributions [27]. A more recent complete genome sequence of the *A. baumannii* strain ACICU (available under accession no. NZ_CP031380.1 [43]) was used, given that the original genome sequence (accession no. NC_010611.1 [44]) contains several sequencing and assembly errors (Table 1). The *A. baumannii* ACICU genome is 3 919 274 bp long, with 3693 ORFs and a mean GC content of 40.14 %. Likewise, the sequence of the *fbs* gene cluster was retrieved from the more recent version of *A. baumannii* ATCC 17978 genome sequence (accession no. NZ_CP012004.1 [37]), instead of the original one (accession no. NC_009085.1 [45]) (Table 1). The *A. baumannii* ATCC 17978 genome sequence is 3 857 743 bp long, with 3682 ORFs and a mean GC content of 40.07 %.

**Table 1.** Annotation of individual genes of iron-uptake clusters

	Gene	Locus tag*	Gene annotation
	*feoA*	DMO12_RS01455	Ferrous iron transport protein A
***feo***	*feoB*	DMO12_RS01460	Ferrous iron transport protein B
	*feoC*	DMO12_RS01465	Ferrous iron transport protein C
	*exbD*	DMO12_RS08415	Biopolymer transporter ExbD
	*exbD*	DMO12_RS08420	Biopolymer transporter ExbD
	*exbB*	DMO12_RS08425	MotA/TolQ/ExbB proton channel family protein
	*tonB*	DMO12_RS08430	Energy transducer TonB
	*hemTR*	DMO12_RS08435	TonB-dependent receptor
***hemT***	–	DMO12_RS08445	Haem-binding protein
	–	DMO12_RS08450	Haem-binding protein
	–	DMO12_RS08455	Oligopeptidase
	–	DMO12_RS08460	ABC permease
	–	DMO12_RS08465	ABC permease
	–	DMO12_RS08470	ATP-binding protein
	–	DMO12_RS08475	Hypothetial protein
	–	DMO12_RS04685	ECF-sigma factor
	–	DMO12_RS04690	Anti-sigma factor
	*hphR*	DMO12_RS04695	TonB-dependent receptor
***hemO***	*hphA*	DMO12_RS04700	Slam-dependent haemophore
	*hsmA*	DMO12_RS04705	Hemophilin secretion modulator
	–	DMO12_RS04710	TonB-related protein
	–	DMO12_RS04715	Haem oxygenase
	–	DMO12_RS04720	Hypothetical protein
	*basJ*	DMO12_RS13105	Acinetobactin biosynthesis isochorismate synthase
	*basI*	DMO12_RS13110	Acinetobactin biosynthesis phosphopantetheinyl transferase
	*basH*	DMO12_RS13115	Acinetobactin biosynthesis thioesterase
	*barB*	DMO12_RS13120	Acinetobactin export ABC transporter permease/ATP-binding subunit
	*barA*	DMO12_RS13125	Acinetobactin export ABC transporter permease/ATP-binding subunit
	–	DMO12_RS13130	Hypothetical protein
	*basG*	DMO12_RS13135	Acinetobactin biosynthesis histidine decarboxylase
	*basF*	DMO12_RS13140	Acinetobactin biosynthesis bifunctional isochorismatase/aryl carrier protein
	*basE*	DMO12_RS13145	(2,3-dihydroxybenzoyl) adenylate synthase
***bas/bau***	*basD*	DMO12_RS13150	Acinetobactin non-ribosomal peptide synthetase subunit
	*basC*	DMO12_RS13155	Putative histamine N-monooxygenase
	*bauA*	DMO12_RS13160	TonB-dependent siderophore receptor
	*bauB*	DMO12_RS13165	Siderophore-binding periplasmic lipoprotein
	*bauE*	DMO12_RS13170	Ferric acinetobactin ABC transporter ATP-binding protein
	*bauC*	DMO12_RS13175	Ferric acinetobactin ABC transporter permease subunit
	*bauD*	DMO12_RS13180	Ferric acinetobactin ABC transporter permease subunit
	*basB*	DMO12_RS13185	Acinetobactin non-ribosomal peptide synthetase subunit
	*basA*	DMO12_RS13190	Acinetobactin non-ribosomal peptide synthetase subunit
	*bauF*	DMO12_RS13195	Acinetobactin utilization protein
	***entA***	DMO12_RS09220	2,3-dihydro-2,3-dihydroxybenzoate dehydrogenase
	*bfnA*	DMO12_RS08630	Siderophore biosynthesis protein
	*bfnB*	DMO12_RS08635	SidA/IucD/PvdA family monooxygenase
	*bfnc*	DMO12_RS08640	DHA2 family efflux MFS transporter permease subunit
	*bfnD*	DMO12_RS08645	Siderophore achromobactin biosynthesis protein AcsC
	*bfnE*	DMO12_RS08650	LucA/LucC family siderophore biosynthesis protein
***bfn***	*bfnF*	DMO12_RS08655	(2Fe-2S)-binding protein
	*bfnG*	DMO12_RS08660	RraA family protein
	*bfnH*	DMO12_RS08665	TonB-dependent receptor
	*bfnI*	DMO12_RS08670	Hypothetical protein
	*bfnJ*	DMO12_RS08675	PepSY domain-containing protein
	*bfnK*	DMO12_RS08680	Hypothetical protein
	*bfnL*	DMO12_RS08685	Acetyltransferase
	*fbsA*	ACX60_RS04655	AraC family transcriptional regulator
	*fbsB*	ACX60_RS04660	Isochorismate synthase
	*fbsC*	ACX60_RS04665	Isochorismatase family protein
	*fbsD/entA*	ACX60_RS04670	2,3-dihydroxybenzoate-2,3-dehydrogenase
	*fbsE*	ACX60_RS04675	Peptide synthetase
	*fbsF*	ACX60_RS04680	Peptide synthetase
	*fbsG*	ACX60_RS04685	Peptide synthetase
	*fbsH*	ACX60_RS04690	2,3-dihydroxybenzoate–AMP ligase
***fbs***	*fbsI*	ACX60_RS04695	Alcaligin biosynthesis protein
	*fbsJ*	ACX60_RS04700	Ornithine decarboxylase
	*fbsK*	ACX60_RS04705	Siderophore biosynthesis protein
	TS1	ACX60_RS04710	Transposase
	TS2	ACX60_RS04715	Transposase
	*fbsL*	ACX60_RS04720	Phosphopantetheinyl transferase
	*fbsM*	ACX60_RS04725	Thioesterase
	*fbsN*	ACX60_RS04730	TonB-dependent siderophore receptor
	*fbsO*	ACX60_RS04735	MFS transporter
	*fbsP*	ACX60_RS04740	Side-tail fibre protein
	*fbsQ*	ACX60_RS04745	Multidrug transporter MatE

*Locus tags and gene annotations refer to the *A. baumannii* ACICU genome (NZ_CP031380.1), except for the *fbs* gene cluster, which refers to the *A. baumannii* ATCC 17978 genome (NZ_CP012004.1).

### Search for iron-uptake gene clusters in *A. baumannii* genomes

The selected compilation of *A. baumannii* genome sequences was mined to search for the presence of iron-uptake gene clusters (Table 1), using the blastn data analysis plugin tool provided in the PubMLST database. Genes were considered to be present when E-value <e^−10^ and >85 % identity across at least 90 % of the sequence length. Three genes showed % identity value under the selected threshold in several isolates: *hemTR* (DMO12_RS08530), *bauA* (DMO12_RS13270) and *bfnH* (DMO12_RS08760), for which blast lower stringency was applied (≥65 % identity). When all the genes of a cluster were found in an individual isolate, it was assumed that the isolate carried a given iron-uptake cluster. Due to the frequent presence of draft genomes, it was plausible that some genes were split into multiple contigs, escaping detection according to the above criteria. Therefore, when a single isolate lacked only a few genes within a cluster, the presence of the missing gene(s) was searched for at the boundaries of the contig (i.e. in the first/last 200 nucleotides). Moreover, the presence of insertion sequences (ISs) was verified using the IS finder tool (https://isfinder.biotoul.fr/). All those genes that were found, even partially, at the edge of a contig were considered to be present in the genome, as well as all those in which insertion or deletion events were detected. This stringency level does not allow us to infer whether individual genes are functional or not.

### Phylogenetic analysis

Concatenated gene sequences from *feo*, *hemO*, *hemT*, *bas/bau* and *bfn* clusters were extracted from all *A. baumannii* isolates and aligned using MAFFT v7.48 [46]. Poorly aligned sequences were manually removed from the multiple sequence alignment. These included genes with insertion/deletion (indel) events or interrupted genes that were found at the boundaries of a contig. Phylogenetic trees were estimated using the neighbour-joining method, and the robustness of the phylogenetic trees was statistically tested with a bootstrap of 1000 replicates, using Geneious Prime software (Biomatters Ltd, Auckland, New Zealand). Trees were visualized in iToL [47]. The presence of genomic islands (GIs) was predicted using IslandViewer 4 [48]. All genomes were annotated using Prokka v1.14.6 with default parameters [49] and then core genome analysis was performed on two separate groups using Panaroo v1.2.10 [50]: the first one including the whole dataset, and the second one limited to 309 ST2 genomes in which the *hemO* gene cluster was detected. Then multiple sequence alignment was performed using Panaroo v1.2.10 on 2062 core genes from the entire dataset and on 2905 core genes from 309 ST2 isolates. SNP-site was used to extract single-nucleotide polymorphisms (SNPs) from the multiple sequence alignments [51] and the trees were inferred using FastTree v2.1 [52]. To quickly estimate the reliability of each split in the tree, FastTree computes local support values with the Shimodaira–Hasegawa test with 1000 resamples.

### Analysis of the *hemO*-flanking regions in *A. baumannii*

To analyse the genomic context of the *hemO* gene cluster, an alignment of the three upstream and three downstream genes of the cluster was generated for *hemO-*positive *A. baumannii* genomes, using Clustal Omega [53]. A total of 732 and 729 genomes were inspected for the analysis of upstream and downstream regions, respectively, since contigs containing incomplete flanking regions were excluded from the alignment. The alignment was visualized using Geneious Prime software (Biomatters Ltd, Auckland, New Zealand).

In isolates lacking the *hemO* gene cluster, the intergenic region between *hemO*-flanking genes, inferred by previous analysis of the *hemO* genomic context, was retrieved from 303 isolates, and aligned with Clustal Omega [53] to generate a WebLogo representation (http://weblogo.threeplusone.com/).

### The *hemO* gene cluster in species other than *A. baumannii*

To detect the presence of the entire 9451 bp *hemO* gene cluster in the genus *Acinetobacter *, the blastn data analysis plugin tool provided in the PubMLST database was used. blastn searches were performed on all *Acinetobacter* species without applying any filtering parameters. A complete *hemO* cluster was considered present when the alignment length exceeded 9000 bp. The *hemO* clusters were retrieved from all positive isolates and aligned using MAFFT v7.48 [46]. Phylogenetic trees were generated using the neighbour-joining method, and the robustness was statistically tested with a bootstrap of 1000 replicates, using Geneious Prime software (Biomatters Ltd, Auckland, New Zealand). The tree was visualized in iToL [47].

## Results and discussion

### Retrieval of *A. baumannii* genomes and population structure analysis

When this study was initiated, the PubMLST dataset of *A. baumannii* comprised 1794 genomes. Of these, only 1071 genomes met the selection criteria described in the Methods section. As expected, the dataset was biased towards ST2 isolates, which are epidemiologically prevalent [10] and comprise 41 % of the total dataset, followed by ST1 (6 %), ST10 (4 %), ST1550 (3 %), ST25 (2 %) and ST79 (2 %) (Fig. S3).

### Iron-uptake gene cluster combinations

**Fig. 2.**
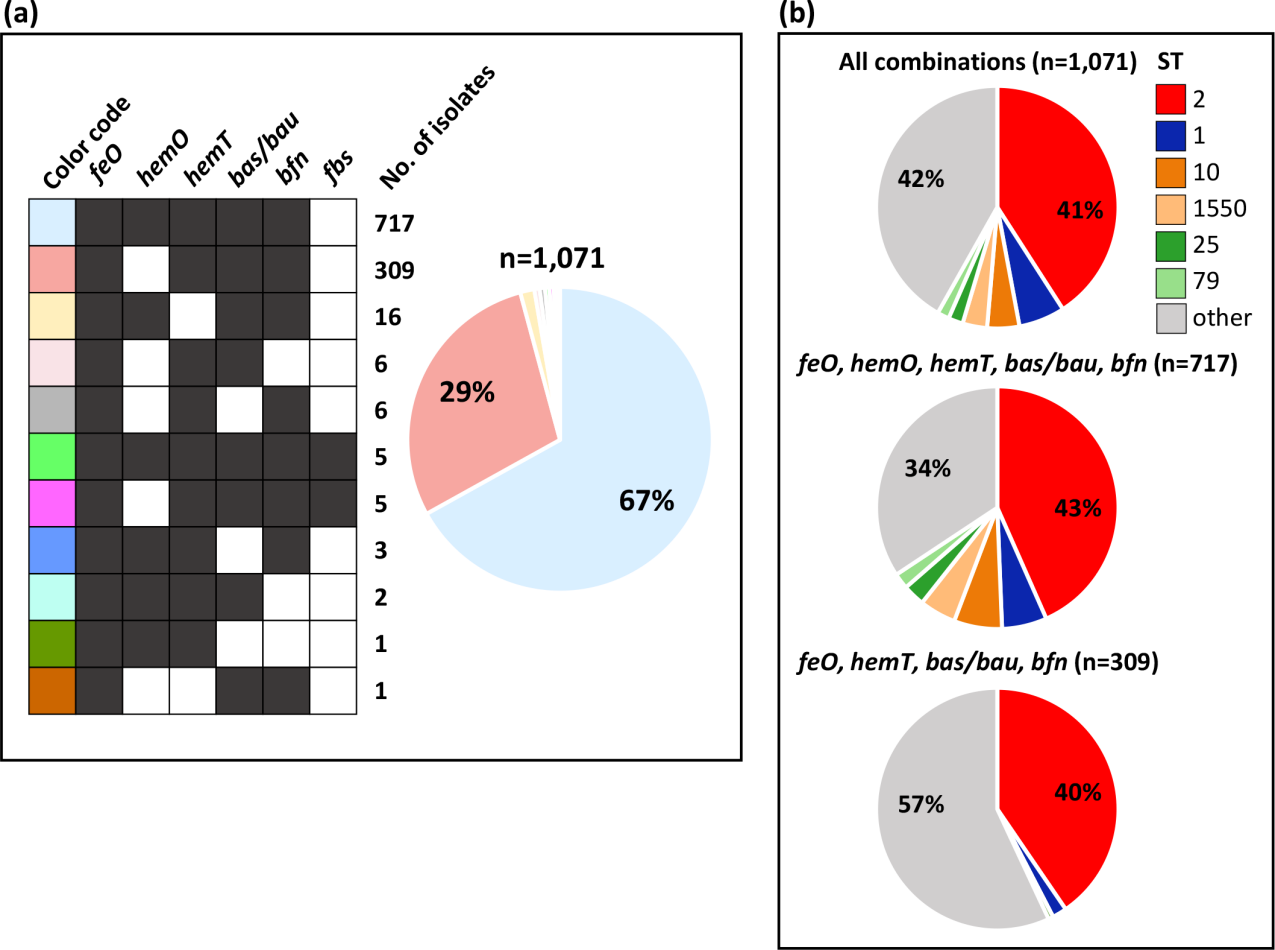
Distribution of iron-uptake gene clusters among 1071 *A *. *baumannii* isolates. (a) The colour code designates each iron-uptake cluster combination; black and white squares denote the presence or absence of the iron-uptake cluster, respectively. The number of positive isolates for each combination is provided. The pie chart on the right shows the relative abundance of each combination according to their colour codes. (**b)** Sequence type distribution of the two most frequent iron-uptake gene cluster combinations, namely isolates lacking only *fbs* (*n*=717) or both *fbs* and *hemO* (*n*=309), relative to all combinations (*n*=1071).

In agreement with previous studies conducted on a smaller scale [22, 26, 27, 37], *feo*, *hemT*, *bfn* and *bas*/*bau* gene clusters were highly prevalent across the dataset (>98 % of isolates), whereas the additional *hemO* haem-uptake cluster was detected in a smaller portion (69 %), and the *fbs* cluster was extremely rare (1 %) (Fig. S4; Table S1). Indeed, most of the isolates (*n*=717; 67 %) carried all clusters except *fbs*, and 309(29 %) carried all clusters except *fbs* and *hemO* (Fig. 2a), implying that the vast majority of *A. baumannii* isolates (*n*=1053; 98 %) carried both the acinetobactin (*bas/bau*) and baumannoferrin (*bfn*) siderophore clusters, and more than half (*n*=728; 68 %) both *hemT* and *hemO* haem-uptake clusters (Fig. 2a). The ST distribution was determined for the most frequent combinations of iron-uptake gene clusters. The ST distribution of isolates carrying all clusters except *fbs* (*n*=717) grossly matched the ST distribution of the whole dataset (*n*=1071), whereas isolates carrying all clusters except *fbs* and *hemO* (*n*=309) were prevalent among less frequent STs (Fig. 2b). Overall, the majority of isolates belonging to the prevalent STs carry all iron-uptake clusters except *fbs*, reflecting the functional versatility of *A. baumannii* iron uptake capability.

### Distribution of siderophore gene clusters across *A. baumannii* clones

**Fig. 3.**
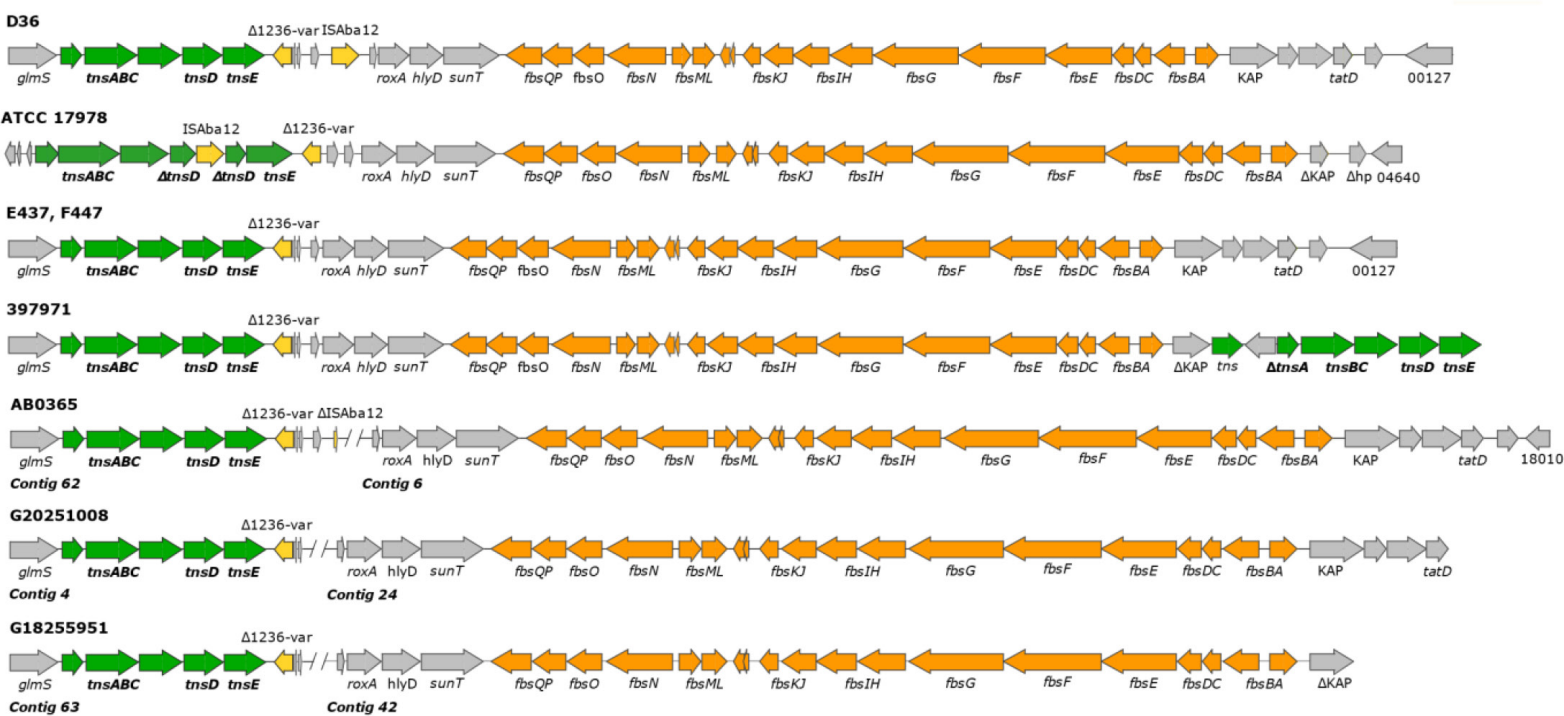
Genetic structure of transposons carrying the fimsbactin(s) gene cluster. Comparison of transposon Tn*6171* in *A. baumannii* strain D36 to transposons from strains ATCC 17978 (ID: 2240), E437 (ID: 6116), F447 (ID: 6117), 397 971 (ID: 2978), AB0365 (ID: 5007), G20251008 (ID: 6582) and G18255951 (ID:6577). The arrows indicate the extent and orientation of genes and ORFs, with genes belonging to the *fbs* cluster in orange, insertion sequences in yellow, transposons in green, and all other genes in grey. The double slash denotes an interruption between two different contigs.

The most important siderophore gene cluster in *A. baumannii* is that responsible for biosynthesis of acinetobactin [29], which is almost ubiquitous (99.0 % of the isolates) across the dataset (Figs 2 and S4). The genomes of only 10 isolates did not contain a complete acinetobactin cluster, including the *A. baumannii* strain SDF genome [54] (Table S1). In 14 isolates indels were observed in the *bas/bau* gene cluster, specifically six in *bauD*, six in *bauE,* one in *basJ* and one in *basD* (Table S2). The *entA* gene was not detected in 13 isolates (1.2 %), including 11 isolates carrying the entire *bas/bau* gene cluster (Table S1). Since *entA* is essential for the biosynthesis of the acinetobactin precursor DHBA [31], its absence suggests that these isolates are unable to produce acinetobactin. The only exception is *A. baumannii* ATCC 17978, which carries an *entA* homologue in the *fbs* gene cluster. In addition to acinetobactin, most *A. baumannii* isolates (99.8 %) also carried the baumannoferrin siderophore cluster [27]. Eight isolates lacked the entire baumannoferrin gene cluster, while only *basJ*, *basF* and *bauF* homologues were detected in *A. baumannii* SDF (Table S1). Gene disruptions due to IS integrations were observed for *bfnB* in two isolates and *bfnA* in one isolate (Table S2). While both acinetobactin and baumannoferrin clusters were detected in nearly all *A. baumannii* genomes, the cluster for fimsbactin biosynthesis and transport was very rare, being detected in only 10 *A *. *baumannii* isolates (Fig. S4), irrespective of STs (Table 2). In most of these isolates, the *fbs* cluster was flanked by transposases (Table 2; Fig. 3), consistent with the previous suggestion that fimsbactin genes have been acquired by horizontal gene transfer [37]. Precisely, the Tn*6171* transposon was detected downstream of the *glmS* gene in the D36 genome (Fig. 3 [37]), and a variant of the same transposon has been found both in the R1B and MRSN 3405 genomes, located in the same chromosomal position (Table 2 [37]). In the genome of ATCC 17978, a closely related transposon, named Tn*6552*, has been identified. Although Tn*6552* shares similarities with Tn*6171*, it also exhibits some variations, including the absence of two genes and an interruption in the *tnsD* gene caused by a copy of IS*Aba12*. Notably, it is not located downstream of the *glmS* gene (Fig. 3 [37]). In E437 and F447 genomes, the structure of the transposon, named Tn*6171*-v3, is very similar to that of Tn*6171*, except for the absence of the IS*Aba12* and the hypothetical protein next to it. Tn*6171*-v3 is precisely inserted in the same chromosomal location as Tn*6171* in both genomes (Fig. 3). In the genome of strain 397 971, the transposon named Tn*6171*-v4 is inserted downstream of the *glmS* gene. While Tn*6171*-v4 is identical to Tn*6171*-v3 on the left side of the *fbs* cluster, on the right side the *tnsABCDE* genes differ due to *tnsA* interruption. Furthermore, an additional *tns* gene is inserted between the interrupted KAP gene and the subsequent gene (Fig. 3). Due to the large number of contigs in the genomes of AB0365, G20251008 and G18255951, the *fbs* cluster and the *tnsABCD* genes were identified on two separate contigs. In particular, the contigs containing the *fbs* cluster are shorter than the total length of the transposon. Although a similar structure was observed on both sides of the *fbs* cluster, arguing for the presence of a transposon, the exact structure could not be determined due to a missing region (Fig. 3). The utilization of more than one siderophore class may facilitate iron acquisition under different environmental conditions and/or iron source availability [29]. However, here we show that the *fbs* cluster is extremely rare in clinical *A. baumannii* isolates (<1 %), while it is more frequent in harmless *Acinetobacter baylyi *, being detectable in 3/14 *A *. *baylyi* genomes available in the National Center for Biotechnology Information (NCBI) database (data not shown). This brings into question the contribution of the *fbs* cluster to *A. baumannii* fitness *in vivo*, and to the emergence of the most successful clonal lineages.

**Table 2.** Isolates carrying the fimsbactin(s) gene cluster

Isolate ID	Isolate name	ST (MLST Pasteur)	Country	Year	Transposon	Reference
3070	D36	81	Australia	2008	Tn6171	[34]
2252	R1B	1	Saudi Arabia	2011	Tn6171*	[34]
2283	MRSN 3405	94	USA	Unknown	Tn6171*	[34]
2240	ATCC 17978	437	France	1951	Tn6552	[34]
6116	E437	1853	Israel	2016	Tn6171-v3	This study; Fig. 3
6117	F447	1853	Israel	2016	Tn6171-v3	This study; Fig. 3
2978	397 971	499	USA	Unknown	Tn6171-v4	This study; Fig. 3
5007	AB0365	94	Singapore	Unknown	na	This study; Fig. 3
6582	G20251008	2	India	2019	na	This study; Fig. 3
6577	G18255951	623	India	2016	na	This study; Fig. 3

*Includes an extra 8 bp repeat unit in the region between IS1236-*var* and ISAba12. na, not assigned. The exact structure of the transposon could not be determined, because *tnsABCDE* and the *fbs* cluster map in different contigs.

### Distribution of haem-uptake gene clusters across *A. baumannii* clones

**Fig. 4.**
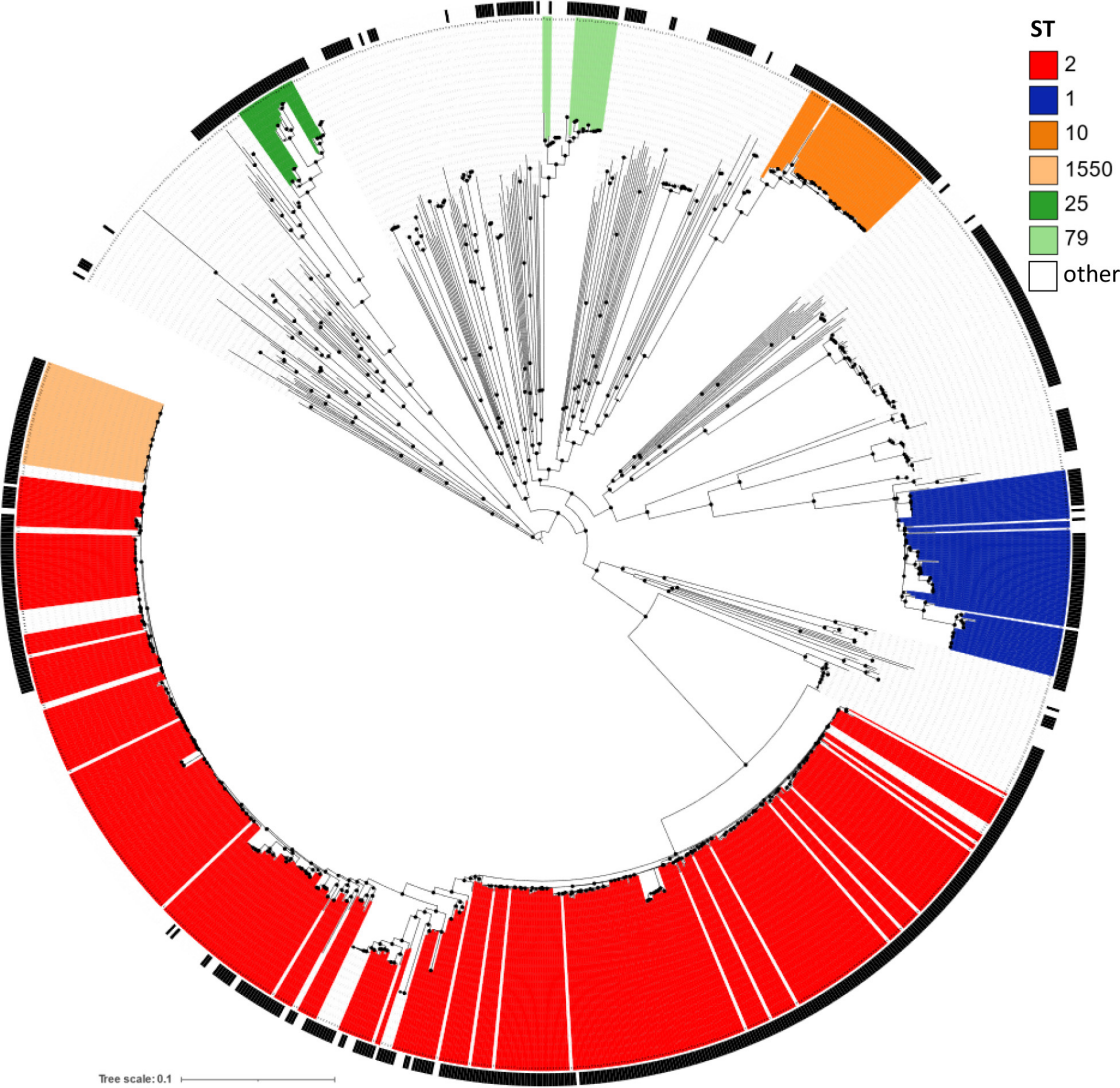
Distribution of the *hemO* gene cluster according to the core genome-based phylogeny of 1071 *A *. *baumannii* strains. The tree was constructed from core genome SNP analysis of 1071 strains from different STs, as shown in the colour code legend. The presence of the *hemO* cluster in the dataset is indicated by the black ring surrounding the tree. Internal node support was determined using the Shimodaira–Hasegawa test, with values ≥80% indicated by black-filled circles. The tree is midpoint rooted, and the scale bar represents the expected number of substitutions per site.

Of the two gene clusters potentially involved in haem uptake, *hemT* was widespread in the dataset, being present in 98.4 % of the isolates. In 16 isolates from the same BioProject (PRJNA613847) no genes of the *hemT* cluster were detected. Of note, these *hemT*-negative isolates originated from Brazil, belonged to ST1, and showed close phylogenetic relationships [55] (Table S1 and Fig. 4). One isolate (ID: 3878) lacked the tandemly arranged *exbD* genes of the *hemT* cluster (Table S1). Major indels (>500 bp) in the *hemT* cluster were detected in five isolates. In detail, two ST79 isolates and one ST2 isolate carried a 2654 bp and a 733 bp insertion in the gene coding for an oligopeptidase (DMO12_RS08455), respectively, one ST2 isolate had a 1179 bp insertion in the gene coding for a putative haem-binding protein (DMO12_RS08445), while a 529 bp deletion in the *exbB* gene was detected in one ST1197 isolate (Table S2).

The *hemO* cluster functions as a high-affinity haem-acquisition system, and it has been suggested to be critical for *A. baumannii* dissemination and/or survival in the blood [38, 56]. The *hemO* cluster was detected in 69 % of the isolates (Fig. S4). Indels in the *hsmA* gene were detected in two isolates (Table S2). While the *hemO* cluster is unevenly spread among isolates, it is prevalent among the most successful clones (Fig. 4). It was detected in 91 % of ST1, 71 % of ST2, 100 % of ST25 and 88 % of ST79 strains. Although it has been argued that haem is important for *A. baumannii* physiology and virulence, it must be considered that this pathogen has evolved several strategies for acquiring diverse forms of iron during infection, so that haem uptake could be useful but not strictly necessary under certain conditions. On the other hand, the prevalence of *hemO* in the most successful *A. baumannii* lineages suggests that it is beneficial to adapt within the host and utilize haem sources, thereby contributing to *in vivo* fitness.

### Intra-species evolution of iron-uptake clusters

**Fig. 5.**
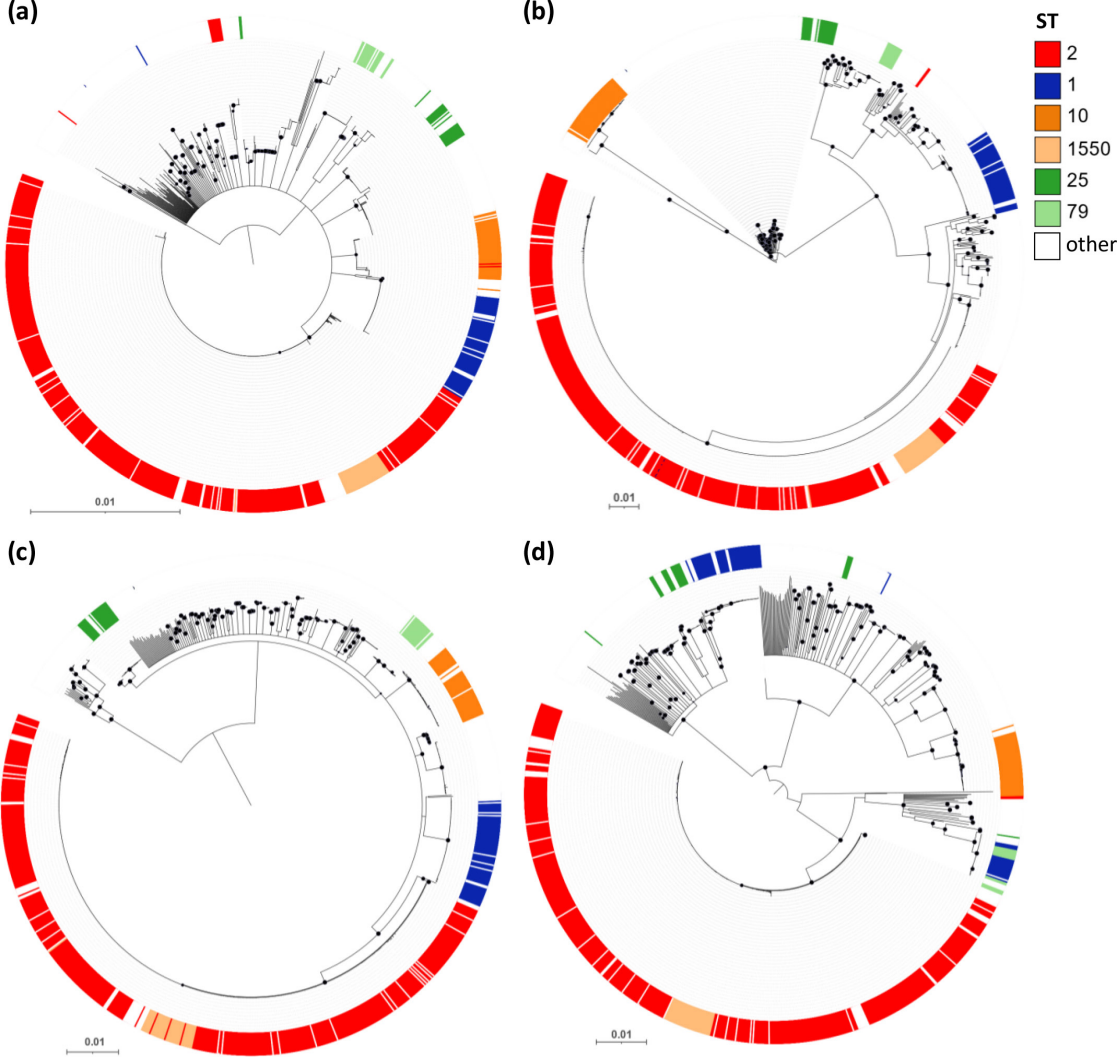
Evolution of the *feo, hemT, bfn* and *bas/bau* iron-uptake gene clusters in *A. baumannii *. Neighbour-joining trees have been generated by the alignment of the concatenated sequences of (**a**) the *feo* gene cluster from 1068 *A *. *baumannii* strains; (**b**) the *hemT* gene cluster from 1013 *A *. *baumannii* strains; (**c**) the *bfn* gene cluster from 1036 *A *. *baumannii* strains; (**d**) the *bas/bau* gene cluster from 1030 *A *. *baumannii* strains. STs are differentiated according to their colour code, as indicated in the key legend. Black-filled circles at the nodes denote bootstrap values ≥80 % (1000 replicates). The trees are midpoint rooted and the scale bars represent the expected number of substitutions per site.

The uneven distribution of some iron-uptake gene clusters among the different *A. baumannii* lineages and some genetic variations observed for almost all of them pose the question of whether – and to what extent – iron-uptake gene clusters evolved along with the differentiation of individual lineages. The possibility exists that some clusters underwent recombination events between isolates belonging to different lineages, or they were either lost or acquired, as could be the case with the *hemO* gene cluster. To address these issues, phylogenetic trees were constructed with the concatenated gene sequences for all iron-uptake gene clusters, except *fbs*. Remarkable phylogenetic congruence was observed for *feo, hemT, bfn* and *bas/bau* gene sequences (Fig. 5), suggesting vertical inheritance and coevolution of these gene clusters along with the evolutionary history of the STs, with rare recombination events affecting the loci. It should be noted that ST1550 isolates clustered within the ST2 clade in all trees, being a single locus variant of ST2.

More in-depth analysis of the evolution of *feo*, *hemT*, *bfn,* and *bas/bau* systems in the most prevalent STs revealed that:

- The *feo* gene cluster from ST1550, ST10, and ST79 showed 100 % phylogenetic congruence with STs. Minor incongruences were observed for a small subset of *feo* gene clusters, including 12 from ST2 (2 %), 2 from ST25 (8 %) and 1 from ST1 (2 %), which were intermingled across the tree (Fig. 5a).
- In the *hemT* phylogeny, all but two (1 %) ST2 isolates grouped together (Fig. 5b). Robust phylogenetic congruence (100 %) was also observed for ST1, ST1550, ST10 and ST79 isolates. Intriguingly, the *hemT* gene cluster from ST10 showed elevated phylogenetic distance and clear separation from other *hemT* clades (Fig. 5b). A blastn search of the *hemT* cluster from an ST10 isolate (Ab04-mff; ID: 3069) revealed the closest similarity (96 %) with the *A. pittii* homologue (GenBank accession nos AP021936.1 and CP028574.2), possibly reflecting genetic exchange and recombination between the *hemT* regions in these closely related species.
- The *bfn* phylogeny showed 100 % congruence for the most frequent STs (Fig. 5c).
- The *bas/bau* tree showed 100 % phylogenetic congruence for ST2, ST1550, ST10 and ST79, but not for ST1 and ST25 (Fig. 5d). Indeed, ST1 isolates were separated into two clusters based on the *bas/bau* phylogeny; almost half of them (45 %) formed a clade with ST79, while the rest (55 %) clustered apart in a single clade (Fig. 5d). All ST1 isolates and most ST79 isolates (10 out of 12) in the ST1/ST79 clade belong to the same BioProject (ID: PRJNA613847) and originate from various sites in Brazil [55], the country from which most ST79 isolates come from (Dataset S1). This suggests that the strains in this clade share a common evolutionary history, as inferred from the *bas/bau* gene cluster phylogeny. Most of the *bas/bau* clusters from ST25 isolates (70 %) form a single clade, while the rest appear intermingled across the tree (Fig. 5d).

**Fig. 6.**
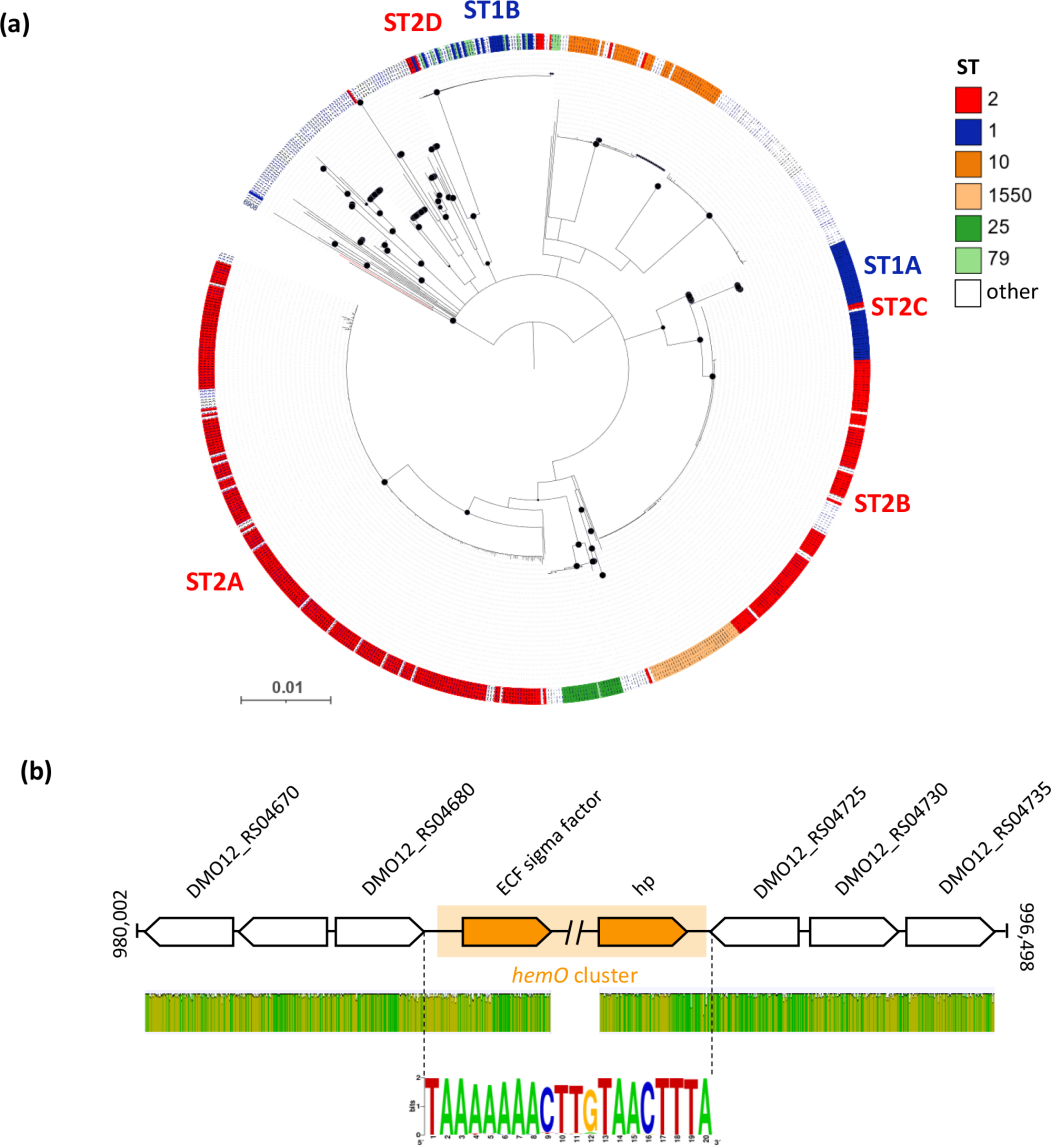
Evolution of the *hemO* clusters and genetic configuration of the *hemO* region in *A. baumannii *. (a) Neighbour-joining tree based on the alignment of concatenated sequences of the *hemO* gene cluster retrieved from 736 *A *. *baumannii* strains. STs are differentiated according to their colour code, as indicated in the key legend. Black-filled circles at the nodes denote bootstrap values ≥80 % (1000 replicates). The tree is midpoint rooted, and the scale bar represents the expected number of substitutions per site. The SDF branch is shown in red. (b) (top) The genomic context of the *hemO* gene cluster in *A. baumannii* ACICU. The orange-shaded region defines the boundaries of the *hemO* gene cluster, spanning from the ECF sigma to the hp gene (orange). The double slash denotes a region of *hemO* that is not shown. The gene map is not to scale. (b) (middle) Level of sequence identity of the *hemO*-flanking region among 732 and 729 *hemO*-posive genomes, including 3 upstream and 3 downstream genes, respectively. Green indicates 100 % identity, greeny-brown at least 30 % identity and red below 30 % identity. (b) (bottom) Consensus sequence of the 20 nt region between the *hemO*-flanking genes (DMO12_RS04680 and DMO12_RS04725, relative to the *A. baumannii* ACICU nomenclature) in 303 isolates lacking the *hemO* gene cluster, generated by the WebLogo representation (http://weblogo.threeplusone.com/ accessed January 2023). The first triplet (TTA) is the stop codon of the upstream gene (DMO12_RS04680), while the last triplet (TAA) is the stop codon of the convergent downstream gene (DMO12_RS04725). The height of the total stack indicates the degree of sequence conservation at a given position.

In contrast to all other clusters, the *hemO* gene cluster phylogeny showed a weak correlation with the STs. It delineates four groups for ST2 isolates, two major (ST2A and ST2B) and two minor ones (ST2C and ST2D) (Fig. 6a). While ST2A is a separate clade, ST2B branches with most of the ST1 isolates (ST1A group, 36 isolates). The *hemO* cluster from two ST2 isolates (ST2C) branched together in the ST1A group (Fig. 6a). The remaining 17 *hemO* clusters from ST1 are grouped in the ST1B clade, along with those from ST79 and ST2 (ST2D group, 7 isolates). Intriguingly, all ST1B and ST79 isolates belong to the same BioProject (ID: PRJNA613847), and originate from Brazil (Dataset S1), showing a similar evolutionary pattern to that observed in the *bas/bau* tree.

Overall, the robust phylogenetic congruence observed for *feo, hemT, bfn* and *bas/bau* gene sequences (Fig. 5) suggests vertical inheritance and coevolution of these gene clusters, along with the evolutionary history of the STs, with rare recombination events affecting some loci. On the other hand, the *hemO* cluster was unevenly distributed in *A. baumannii* and showed limited phylogenetic congruence with STs, implying that the presence or absence of this cluster may have resulted from different acquisition (integration) or loss (excision) events that occurred at different times during *A. baumannii* evolution.

### Evolution of the *hemO* gene cluster

The core genome SNP phylogeny of *hemO*-positive ST2 isolates shows that microevolution of the *hemO* cluster is consistent with the division of the ST2 isolates into two major subgroups, namely ST2A and ST2B (Figs 6a and S5). This suggests that the ancestral *hemO* cluster evolved according to the phylogeny of the isolates while being occasionally lost by some of them. However, it cannot be ruled out that the *hemO* gene cluster was acquired by multiple independent events and then inherited by the descendant population. In the latter case, the *hemO* cluster should be endowed with structural features enabling its insertion on the *A. baumannii* chromosome. To test this hypothesis, the flanking regions of the *hemO* cluster, including the three downstream and three upstream genes, were inspected in more detail. An alignment performed using 744 *hemO*-positive genomes revealed that the upstream region is highly conserved in 732 genomes (98 %), while the downstream region is conserved in 729 genomes (98 %) (Fig. 6b). Therefore, in nearly all *hemO*-positive genomes, the *hemO* gene cluster maps in the same chromosomal location, except for strain SDF, consistent with previous reports [56–58]. On the other hand, in *hemO*-negative isolates, a highly conserved 14 nt sequence was identified in the intervening region between the genes located upstream (DMO12_RS04680) and downstream (DMO12_RS04725) of the *hemO* gene cluster (Fig. 6b). Remarkably, ISs, genomic islands, transposons and recognition sequences for site-specific recombinases were not detected in the regions surrounding the *hemO* gene cluster, except for the previously observed IS*Aba125* located ~3000 bp upstream of the cluster [27]. Therefore, it is unlikely that the *hemO* gene cluster has been acquired by *A. baumannii* through multiple independent events, since it invariably maps in the same chromosomal location, with no evidence of site-specific recombination signatures. More likely, it has been lost by independent excision events that occurred during *A. baumannii* evolution, as *hemO* consistently follows the phylogeny of clonal groups. However, the molecular mechanisms that have directed the excision of the *hemO* gene cluster remain unpredictable.

**Fig. 7.**
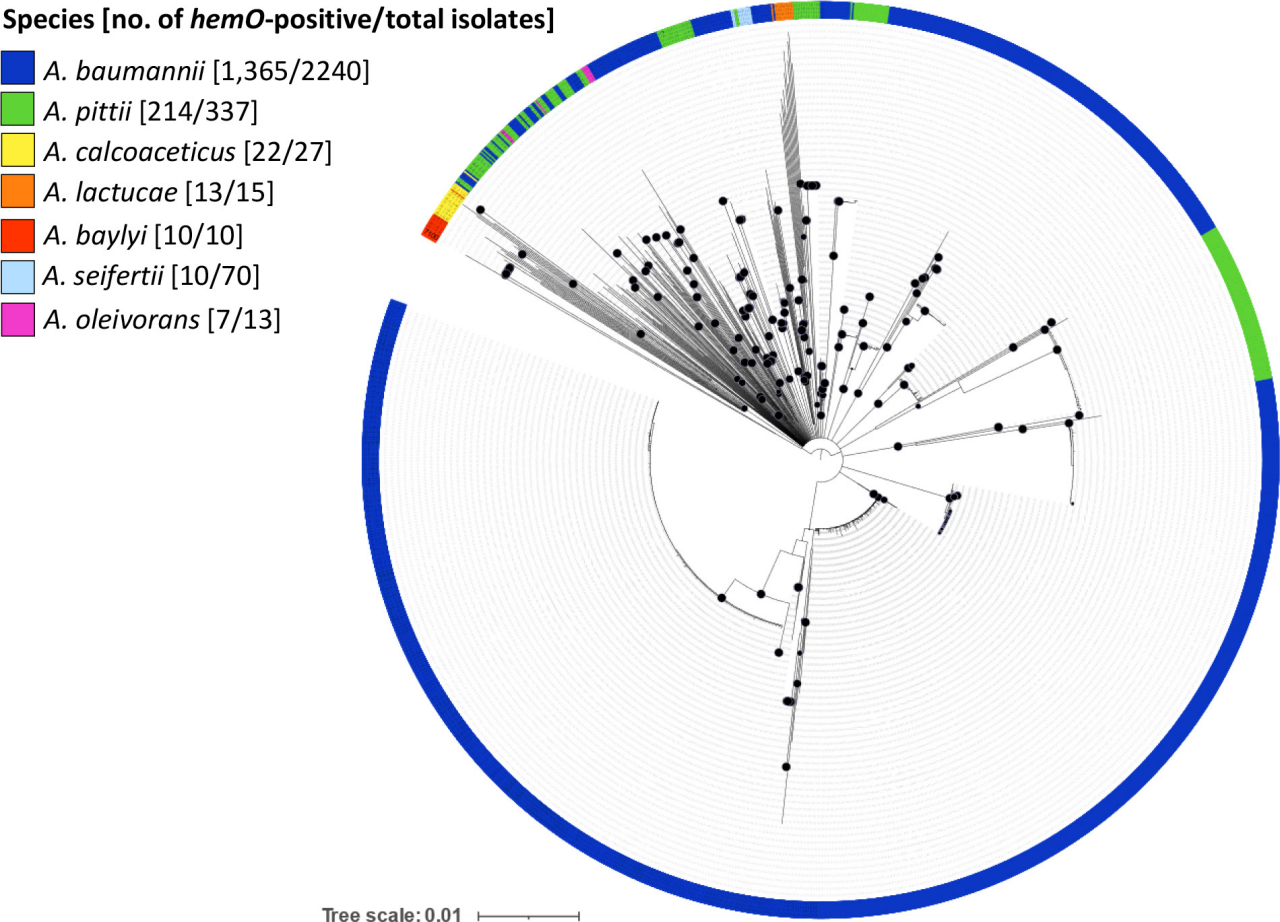
Evolutionary history of the *hemO* gene cluster in genus *Acinetobacter *. Unrooted neighbour-joining tree generated from the sequence alignment of the entire *hemO* gene cluster retrieved from 1641 genomes of different species belonging to the genus *Acinetobacter *. Species are differentiated by colour, as indicated in the key legend. Black-filled circles at the nodes denote bootstrap values ≥80 % (1000 replicates). The scale bar indicates the expected number of substitutions per site.

Intriguingly, the *hemO* gene cluster is unique to the genus *Acinetobacter *, and has no homologue in other bacterial genera. It is harboured, with variable frequency, by *Acinetobacter lactucae *, *Acinetobacter baylyi *, *Acinetobacter calcoaceticus *, *Acinetobacter oleivorans *, *Acinetobacter pittii* and *Acinetobacter seifertii *, besides *A. baumannii* (Fig. 7). It is also present in *A. baumannii* SDF, an aberrant isolate from a human body louse, branching apart in the MLST phylogeny due to massive genome reduction [58]. With the exception of *A. baylyi* and *A. oleivorans* (a species without a validly published name according to The List of Prokaryotic names with Standing in Nomenclature (LPSN; https://lpsn.dsmz.de/species/acinetobacter-oleivorans) [59], all *hemO*-positive *Acinetobacter* species form a single cluster, called the ACB complex, in the core genome-based phylogeny of genus *Acinetobacter* [60]. It is therefore tempting to speculate that the *hemO* cluster was present in the ACB complex ancestor and that it was subsequently lost by some isolates after speciation. Intriguingly, *Acinetobacter nosocomialis* is the only member of ACB complex lacking the *hemO* cluster, which was not found in any of the 224 genomes present in the PubMLST database. This can be explained by an excision event that occurred at the beginning of *A. nosocomialis* speciation, when it started diverging from *A. baumannii* [60]. Moreover, the invariable absence of the *hemO* gene cluster in *A. nosocomialis *, a species that shares its ecological niche with members of the ACB complex, argues against the possibility that the *hemO* gene cluster has been acquired by *Acinetobacter* species through multiple independent events. Conversely, *A. baylyi* could have acquired the *hemO* cluster by genetic exchange with any species of the ACB complex, since this species is particularly prone to horizontal gene transfer [61]. Overall, the phylogenetic tree constructed using the *hemO* region highlights a consistent topology with the phylogeny of the genus *Acinetobacter *, uncovering that the evolution of the *hemO* cluster parallels the evolution of the different *Acinetobacter* species (Fig. 7). This strengthens the hypothesis that the *hemO* cluster was present in the common ancestor of the ACB complex, and subsequently inherited by descendant species.

## Conclusions

The success of *A. baumannii* as a colonizer and a pathogen is partly due to its ability to face the low iron availability imposed by the host [2, 26]. Different iron forms are present in tissues and biological fluids of mammals and birds, so that expression of multiple iron-uptake systems is linked to virulence, reflecting the adaptive potential required to colonize different niches inside the host.

Small-scale *in silico* studies suggested that multiple iron-uptake systems are present in different combinations in genotypically diverse *A. baumannii* isolates [27, 39], motivating our large-scale analysis of the distribution of iron-uptake gene clusters in the global *A. baumannii* population. To this purpose, a dataset of 1071 *A *. *baumannii* genomes representative of the most frequent lineages circulating worldwide was assembled and scrutinized to determine the distribution of iron-uptake systems among different lineages and envisage correlations between the iron-uptake capability and the epidemiological success of the most prevalent lineages. The PubMLST database was used as the source of information, as it provides useful metadata for establishing epidemiological associations. Our results provide evidence that nearly all *A. baumannii* genomes carry ferrous iron (*feo*), acetinobactin (*bas/bau*) and the baumannoferrin (*bfn*) gene clusters, along with the *hemT* cluster for haem uptake. The *hemO* gene cluster, encoding an additional haem-uptake system, was detected in most genomes, whereas the *fbs* gene cluster was extremely rare, and carried by a composite transposable element. In theory, the *hemO* gene cluster should provide a selective advantage *in vivo*, thanks to the efficient utilization of the host’s haemoglobin as an iron source [38, 57, 62]. This hypothesis is partly supported by *hemO* detection in a significant number of *A. baumannii* isolates belonging to the epidemiologically prevalent STs. It is also worth mentioning that *hemO* is conserved in the extensively decayed genome of strain SDF, originally isolated from a haematophagous arthropod [27, 54, 63]. On the other hand, the *hemO* gene cluster was absent in nearly one-third of the *A. baumannii* population, so it cannot account per se for the epidemiological success of prevalent STs. It is plausible that the *hemT* system, whose function and substrate specificity are still elusive at present, replaces *hemO* function under certain conditions. Along the same lines, the occasional occurrence of the *fbs* gene cluster in the dataset denotes the marginal role of the fimsbactin-mediated iron uptake in the emergence and/or spread of the most successful *A. baumannii* lineages.

While the coexistence of multiple iron-uptake systems denotes broad nutritional versatility, the hierarchy by which individual systems contribute to *A. baumannii* fitness is still unclear. Another versatile pathogen, *Pseudomonas aeruginosa *, can adapt its iron-uptake strategy to best fulfil its needs for the metal without spending too much energy. It exploits at least 15 different iron-uptake strategies, relying on 2 endogenous siderophores, and the uptake of haem and xenosiderophores (chelators produced by other bacteria) to save on endogenous siderophore production [64, 65]. Whether *A. baumannii* also senses the presence of chelators in the environment and acquires iron by the most convenient uptake strategy remains to be elucidated. It is worth noting that up to 16 TonB-dependent receptor genes have so far been predicted in *A. baumannii *, in addition to those involved in the uptake of haem (*hemT* and *hphR*), acinetobactin (*bauA*), baumannoferrin (*bfnH*) and fimsbactin (*fbsN*) [22, 27, 66]. It is tempting to speculate that in *A. baumannii* the additional TonB-dependent receptors also contribute to iron uptake via xenosiderophores, through a piracy strategy aimed at economizing on endogenous siderophore production. Such a large repertoire of iron-acquisition systems in *A baumannii* implies an even larger repertoire of possible combinations of iron-acquisition systems, whose expression can be tuned according to the energetic status of the cell, the available iron source(s) and the physicochemical conditions of the growth environment. While the natural substrate specificity of the additional TonB-dependent receptors is still unclear, the *piuA* (DMO12_RS02620) and *pirA* (DMO12_RS05030) xenosiderophore receptors have recently been associated with the transport of cefiderocol, a siderophore–cefalosporin conjugate endowed with good activity on MDR *P. aeruginosa* and *A. baumannii* [67, 68].

Acquisition of novel iron-uptake capabilities via horizontal gene transfer of mobile genetic elements has been associated with the emergence of particularly virulent clones in some pathogenic bacteria [69–73]. However, all iron-uptake gene clusters described in *A. baumannii* are chromosomally encoded and show no evidence of mobilization signatures in their flanking regions, except for the transposon-carried *fbs* gene cluster. Accordingly, *feo, hemT*, *bfn* and *bas/bau* gene clusters showed remarkable congruence with the MLST-based phylogeny, suggesting vertical inheritance and coevolution along with the evolutionary history of individual lineages. The *hemO* gene cluster deserves a separate comment, since it was unevenly detected in the *A. baumannii* population, although invariably mapping in the same chromosomal position without signatures of recent recombination events. The phylogeny of the *hemO* gene cluster in the genus *Acinetobacter* suggests that it was present in the ancestor of the ACB complex, and subsequently inherited by descendant *Acinetobacter* species, before being lost by some strains during evolution, probably influenced by natural selection or genetic drift. While recombination mechanisms that may have directed the loss of the *hemO* gene cluster are still obscure, they appear to be specific, since a highly conserved 14 nt ‘scar’ pattern was observed at the putative site of excision. Several counterselecting factors may have contributed to the loss of the *hemO* gene cluster in *Acinetobacter* sp. For instance, free haem can be toxic to some species due to free iron release by haem-oxygenase [74], an enzyme encoded by the *hemO* gene cluster. It is also possible that *hphR* serves as the outer membrane receptor for recognition by phages or bacteriocins, as it does for TonB-dependent transporters in other species [75–77], implying that the loss of the *hemO* gene cluster may help to protect from potentially lethal agents.

Lastly, given the essential role of iron in *A. baumannii* pathogenicity, iron metabolism is now regarded as a druggable target for the development of novel antimicrobial strategies. In this perspective, iron-uptake clusters that are highly conserved amongst clinical isolates represent attractive candidates for the implementation of novel therapeutics, e.g. siderophore–antibiotic conjugates [41, 78], to combat MDR *A. baumannii *.

## Supplementary Data

Supplementary material 1

Supplementary material 2

Supplementary material 3

Supplementary material 4
