# Supplementary material for: Genomics of Acinetobacter baumannii iron uptake
Source: Microb Genom. 2023 Aug 7;9(8):mgen001080. doi: 10.1099/mgen.0.001080 (PMC10483418; doi:10.1099/mgen.0.001080)
Supplement: Supplementary material 1 [file mgen-9-1080-s001.pdf]

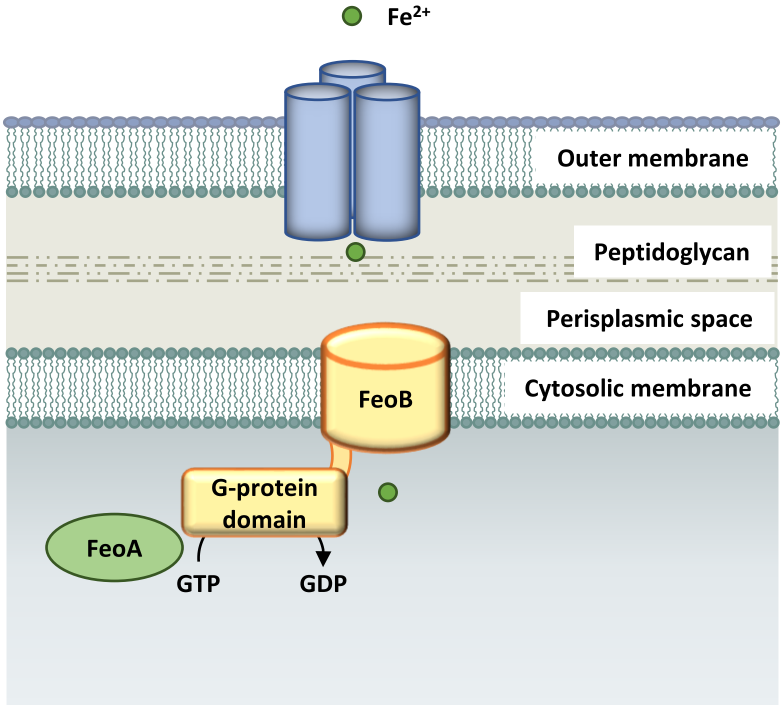

Fig. S1. Schematic representation of Feo system. The Fe^{2+} soluble iron form can diffuse into the periplasm through porins. Fe^{2+} is then transported across the cytoplasmic membrane by FeoB thanks to a GTP-driven active transport process. The FeoB protein contains a G-protein domain at its N-terminus and C-terminal integral-membrane domain is predicted to consist of eight transmembrane α-helices. FeoA is thought to be required for maximal FeoB activity, likely by activating the GTPase function of FeoB.

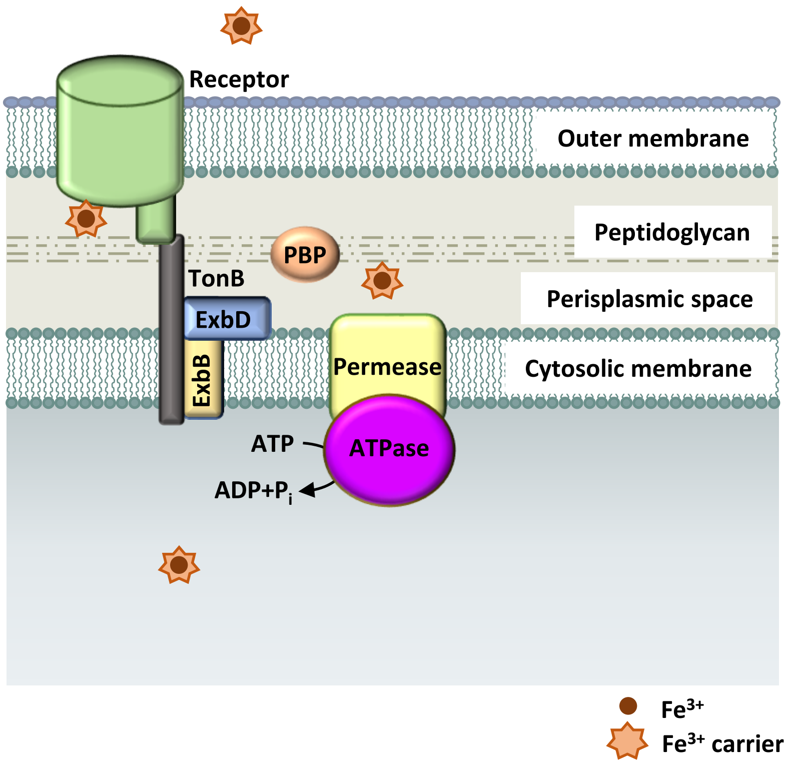

Fig. S2. Schematic representation of TonB/ExbB/ExbD energy-transducing system. Bacterial outer membrane receptors transport the Fe^{3+}-chelator complexes or heme into the periplasm thanks to their interaction with TonB/ExbB/ExbD complex, which transduces the energy of the proton gradient on the inner membrane into conformational changes of the receptors. Once in the periplasm, transport across the inner membrane is facilitated by ABC transport systems.

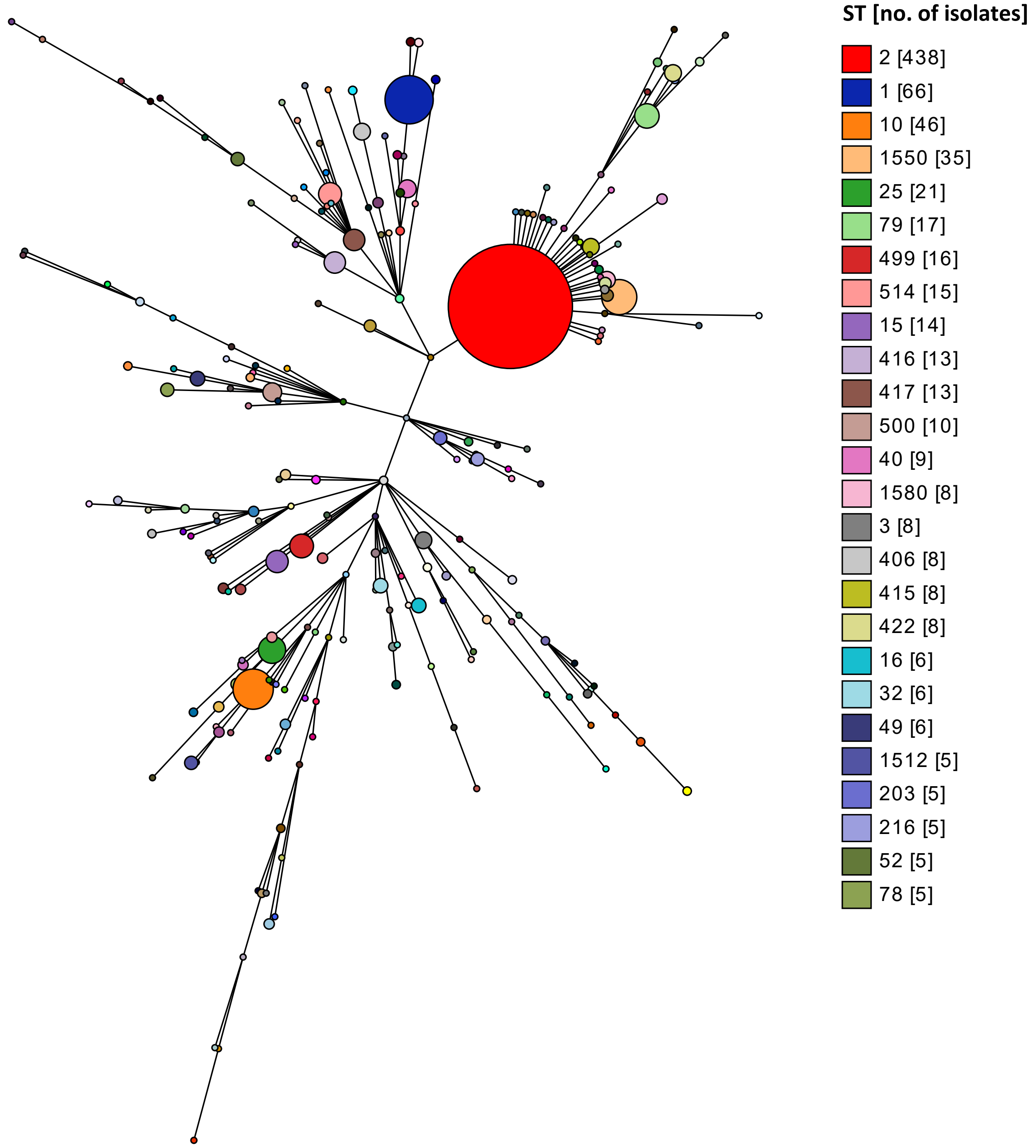

Fig. S3. Population structure of 1,071 *A. baumannii* analyzed by GrapeTre. The size of each node is proportional to the number of strains within that node. Each node represents one of the 227 sequence types (STs) and is color-coded accordingly. The key legend indicates the color codes for each ST represented by more than 5 strains.

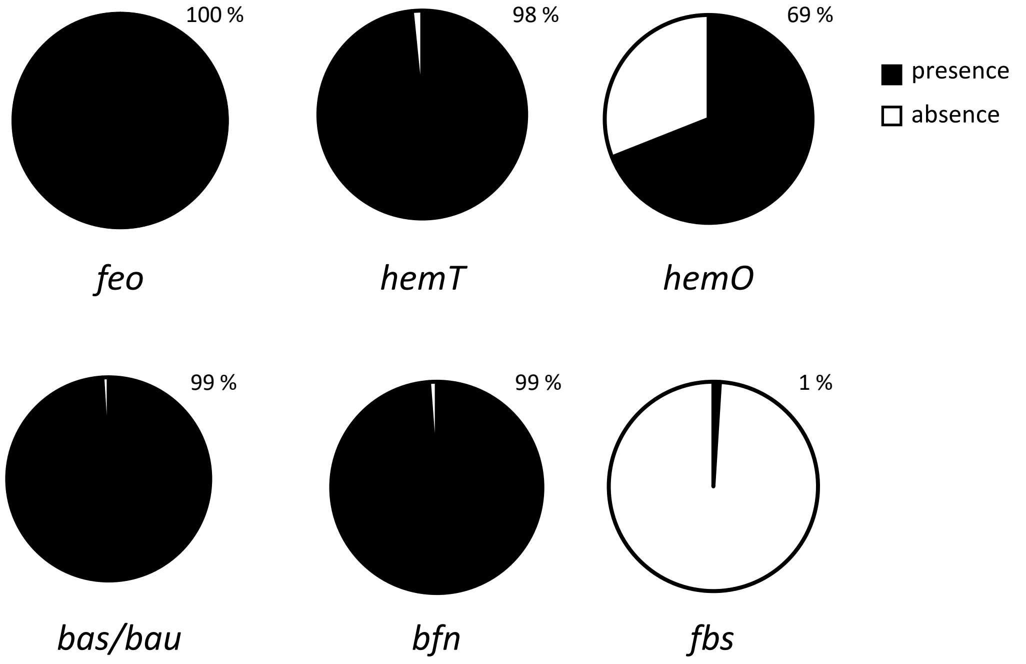

Fig. S4. Occurrence of different iron-uptake gene clusters among 1,071 *A. baumannii* strains. Each pie chart shows the proportion of isolates carrying a given iron uptake gene cluster.

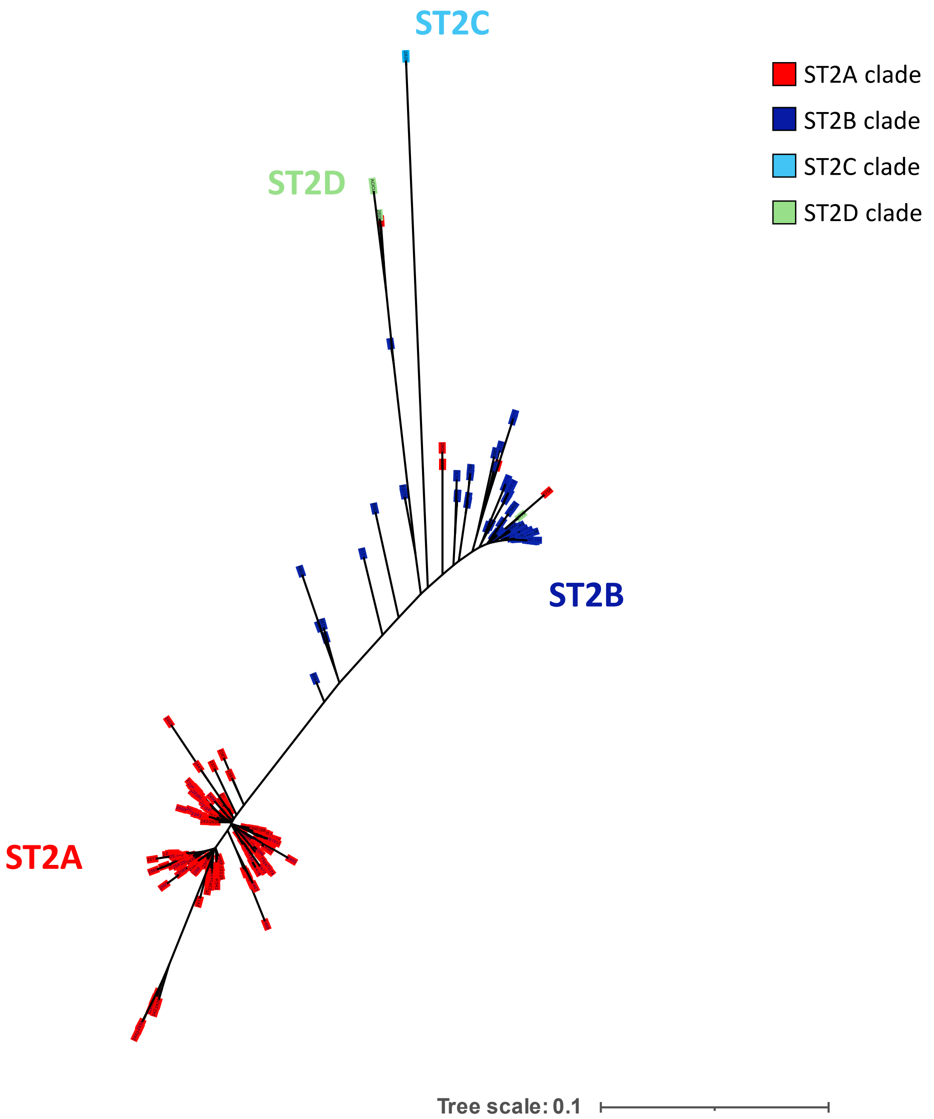

Fig. S5. Core-genome SNPs phylogeny of ST2 isolates carrying the *hemO* gene cluster. Unrooted Neighbor-Joining tree obtained by core-genome SNPs analysis of 309 ST2 isolates carrying the *hemO* gene cluster. The division of the ST2 isolates into two major subgroups, namely ST2A and ST2B, is consistent with the microevolution of the *hemO* cluster (see Figure 6). Support for internal nodes was determined based on the Shimodaira–Hasegawa test. The scale bar indicates the expected number of substitutions per site.
